# Supplementary material for: Biological factors in the synthetic construction of overlapping genes
Source: BMC Genomics. 2021 Dec 11;22:888. doi: 10.1186/s12864-021-08181-1 (PMC8665328; doi:10.1186/s12864-021-08181-1)
Supplement: Supplementary file 1 — Additional file 1. [file 12864_2021_8181_MOESM1_ESM.docx]

**Supplementary Figures**


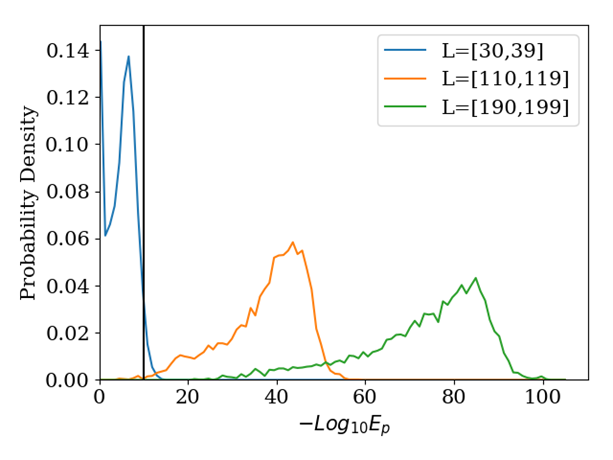

Figure S1: E-value distributions for OLG sequences with different lengths. The black line indicates an e-value of 10^(-10) as used in [3]. The e-value is strongly length dependent, so a BLAST evaluation prohibits a fixed e-value cutoff.


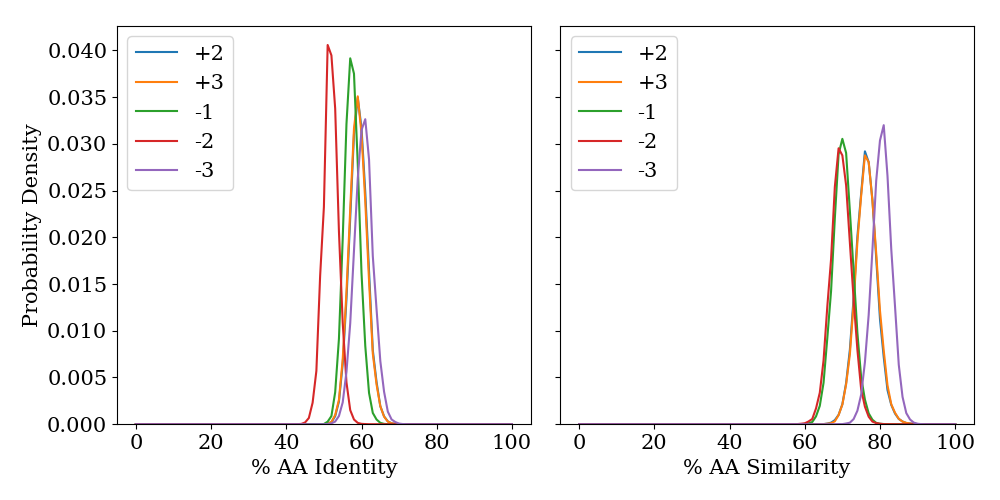


Figure S2: Probability density for different amino acid identities and similarities split by reading frame. The data is calculated from 505.000 OLG pairs. Left: The sequence with the lower identity is representative of the pair. Right: The mean similarity between both OLG sequences represents the pair.


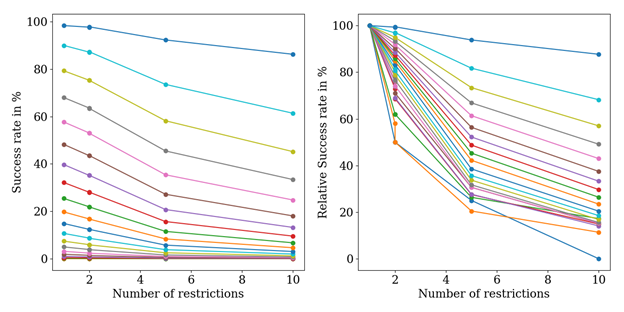

Figure S3: OLG construction success rate as a function of combinatorial restrictions on the SGC. From top to bottom each line represents a 5% higher threshold value, starting with the lowest possible threshold, namely successful OLGs must be at least as good as the worst sequence of its protein family. In order to make different thresholds on the left more comparable, the figure on the right is the same data but every success rate is normalized by the value of the ‘-3’ frame. The linear relationship is gradually lost with higher thresholds.


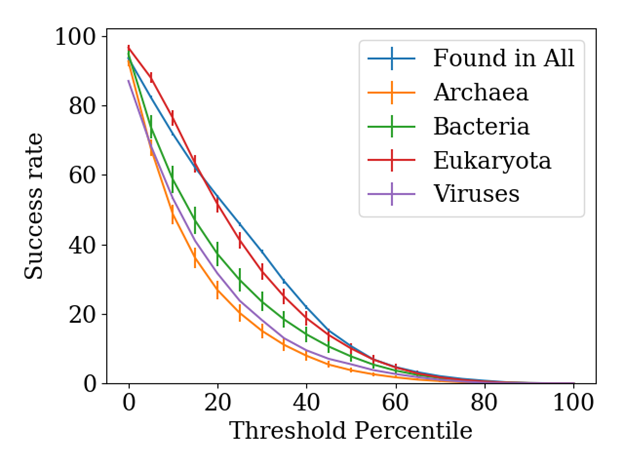


Figure S4: Average success rate as a function of threshold percentile for different taxonomic groups. The vertical bars indicate the standard deviation. Averages and standard deviations are calculated from 20 datasets with 150 sequences each, which are at least 70 amino acids long. Taxonomic differences are mostly independent of the threshold percentile.


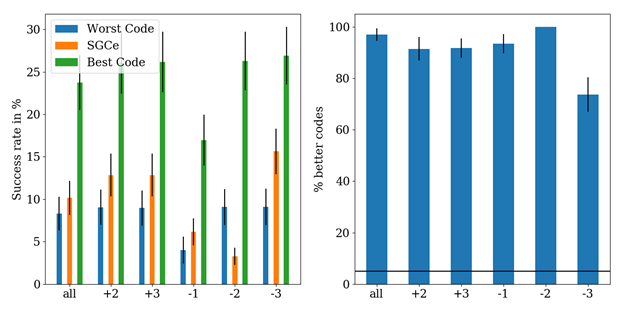


Figure S5: Average Success rate of random codes calculated from 20 sets of 100 genetic codes - each set uses different protein domains; the black bars indicate the standard deviation. Left: Average success rate of the best and worst codes compared to the SGC. Right: Percentage of codes with a higher OLG construction success rate for all reading frames and an average across all reading frames. The SGC is closer to the worst code in this code set than the best and is very suboptimal in this code set.


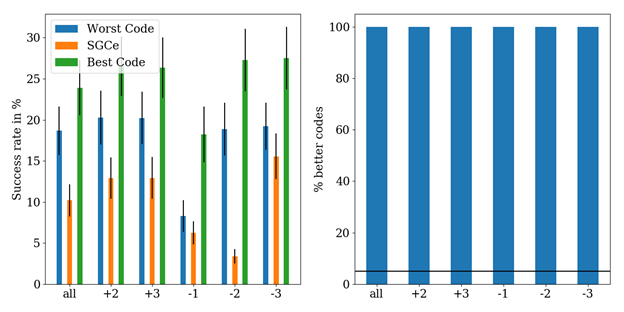


Figure S6: Average Success rate of degeneracy codes calculated from 20 sets of 100 genetic codes - each set uses different protein domains; the black bars indicate the standard deviation. Left: Average success rate of the best and worst codes compared to the SGC. Right: Percentage of codes with a higher OLG construction success rate for all reading frames and an average across all reading frames. The SGC is always much worse than the worst code in the degeneracy code set and therefore a very suboptimal code in comparison.


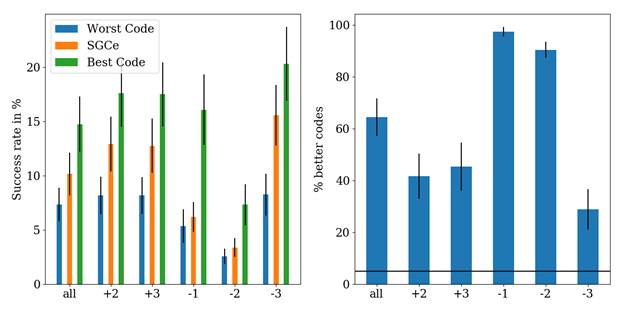


Figure S7: Average Success rate of block codes calculated from 20 sets of 100 genetic codes - each set uses different protein domains; the black bars indicate the standard deviation. Left: Average success rate of the best and worst codes compared to the SGC. Right: Percentage of codes with a higher OLG construction success rate for all reading frames and an average across all reading frames. The SGC is a typical code in this code set.


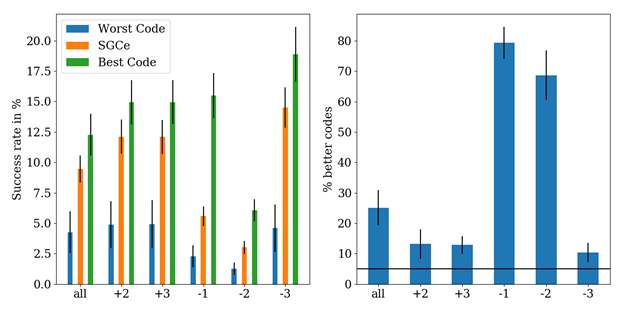


Figure S8: Average Success rate of MR-block codes calculated from 10 sets of 500 genetic codes - each set uses different protein domains; the black bars indicate the standard deviation. Left: Average success rate of the best and worst codes compared to the SGC. Right: Percentage of codes with a higher OLG construction success rate for all reading frames and an average across all reading frames. The SGC is close to the best codes in this code set and almost passes the optimality threshold of 5% (black line).


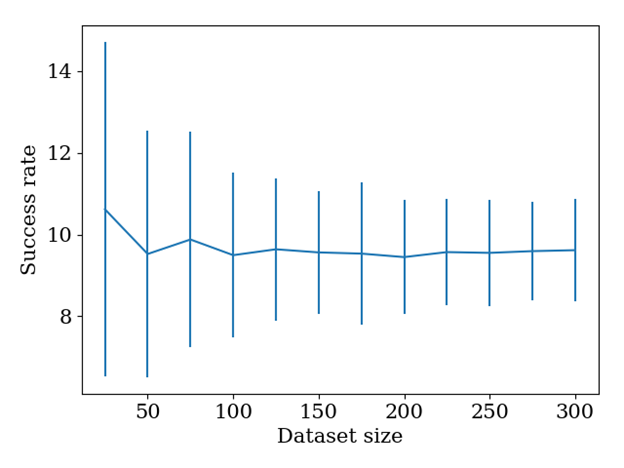


Figure S9: Variation of the average OLG success rate as a function of the number of domains in each dataset. The line indicates the mean value over 100 different sets of domains and the bars indicate the standard deviation. Domains have a minimum length of 70 amino acids and each domain pair is overlapped at one random position. The 50th percentile is used as a threshold for a successful overlap. Using a dataset size of 150 sequences is a good tradeoff between minimizing fluctuations and a computation time.


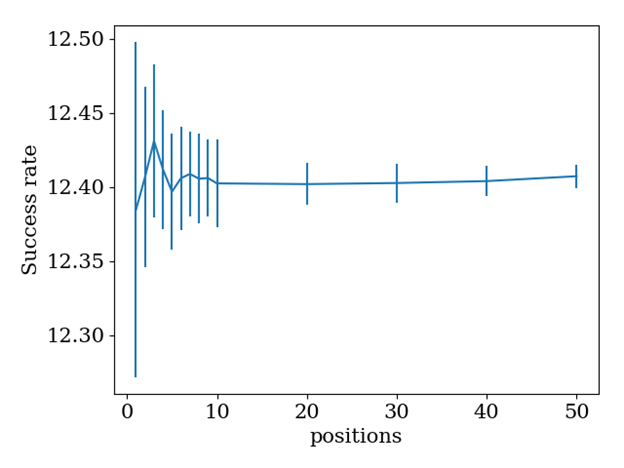


Figure S10: Variation of the average OLG success rate as a function of the number of random overlap positions. The line indicates the mean value over 30 different sets of positions and the bars indicate the standard deviation. 150 randomly chosen domains with a minimum length of 70 amino acids are used as a basis. The 50th percentile is used as a threshold for a successful overlap. Even when only picking one position for each overlap, variations in the resulting success rates are neglectable.


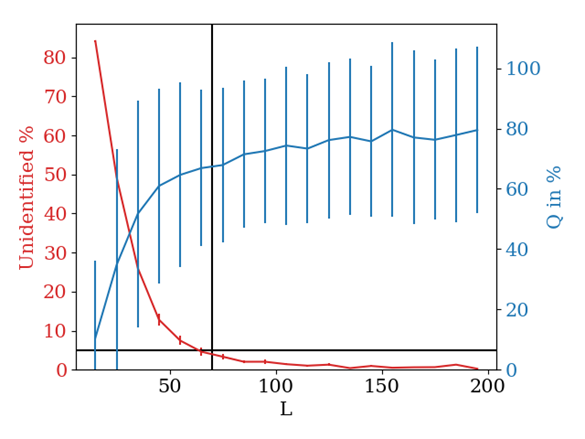


Figure S11: Average quality Q and the number of unidentified sequences of constructed OLG pairs as a function of sequence lengths L. Vertical blue lines indicate two times the standard deviation. The horizontal black line indicates 5% unidentified sequences. The vertical black line indicates the chosen length cutoff of 70 amino acids. The data was calculated from 20 datasets with 150 sequences each. For very small and very large sequences just a few more than 150 sequences exist in the Pfam database so there is a strong overlap between datasets.


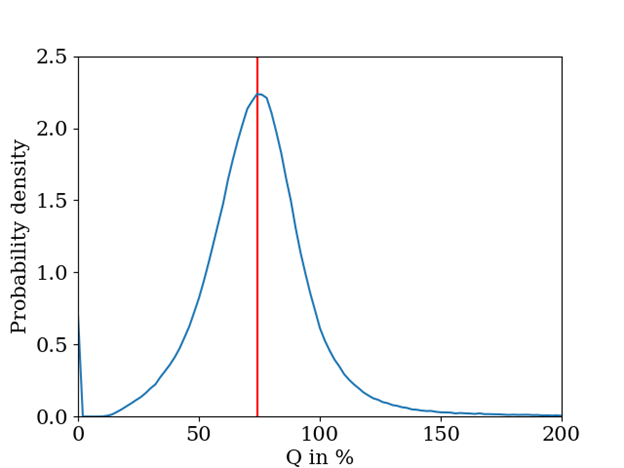

Figure S12: Average Q distribution for constructed OLGs for sequences with 100-109 amino acids. The red line indicates the peak of the distribution at 76%. The average was calculated for 20 datasets with 150 sequences. This represents the typical quality loss due to overlapping random domains.


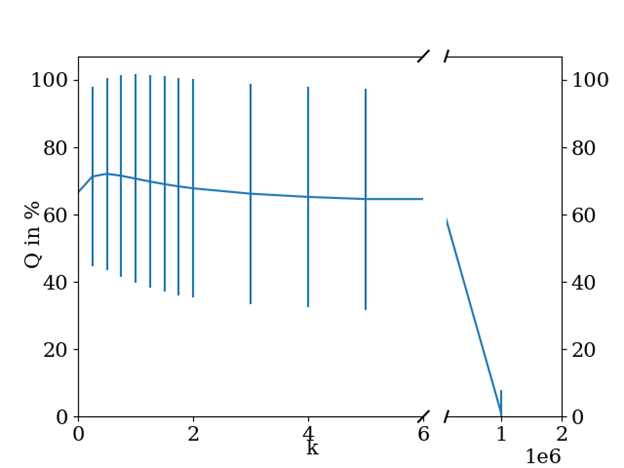


Figure S13: Average Q of OLG pairs as a function of weight strength k. The vertical lines indicate the standard deviation calculated from 20 datasets with 150 sequences of a minimum length of 70 amino acids each. The weights have a weak but positive influence on the OLG construction. For very high weight strengths at each position the sequence with the higher conservation will always maintain its amino acid and the Q goes to zero, indicating that balancing the two sequences is very important.


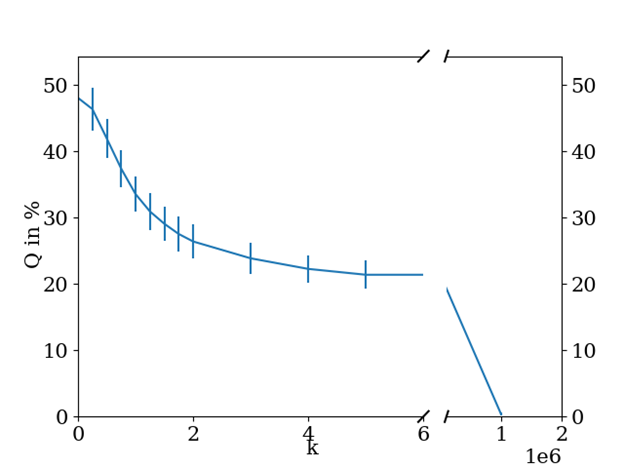


Figure S14: Average percentage of functional OLG pairs as a function of weight strength k when evaluated with BLAST. The vertical lines indicate two times the standard deviation. The data has been calculated from 5 datasets with 100 sequences of length 40-60 amino acids. Introducing weights can only reduce sequence quality in the BLAST evaluation.
